# Health effects of herbicides and its current removal strategies

**DOI:** 10.1080/21655979.2023.2259526

**Published:** 2023-09-25

**Authors:** Rozidaini Mohd Ghazi, Nik Raihan Nik Yusoff, Nurul Syazana Abdul Halim, Ikarastika Rahayu Abdul Wahab, Nurzila Ab Latif, Siti Halimah Hasmoni, Muhammad Abbas Ahmad Zaini, Zainul Akmar Zakaria

**Affiliations:** a Faculty of Earth Science, Universiti Malaysia Kelantan - Jeli Campus, Jeli, Kelantan, Malaysia; b Faculty of Agro-Based Industry, Universiti Malaysia Kelantan Jeli Campus, Jeli, Kelantan, Malaysia; c Department of Biosciences, Faculty of Science, Universiti Teknologi Malaysia, Johor Bahru, Johor, Malaysia; d Department of Chemical Engineering, Universiti Teknologi Malaysia, Johor Bahru, Johor, Malaysia; e Department of Bioprocess and Polymer Engineering, Faculty of Chemical and Energy Engineering, Universiti Teknologi Malaysia, Johor Bahru, Johor, Malaysia

**Keywords:** Herbicide contamination, mode of action, adverse effect, human health, physico-chemical treatment, biological treatment, chemical treatment, sustainable treatment

## Abstract

The continually expanding global population has necessitated increased food supply production. Thus, agricultural intensification has been required to keep up with food supply demand, resulting in a sharp rise in pesticide use. The pesticide aids in the prevention of potential losses caused by pests, plant pathogens, and weeds, but excessive use over time has accumulated its occurrence in the environment and subsequently rendered it one of the emerging contaminants of concern. This review highlights the sources and classification of herbicides and their fate in the environment, with a special focus on the effects on human health and methods to remove herbicides. The human health impacts discussion was in relation to toxic effects, cell disruption, carcinogenic impacts, negative fertility effects, and neurological impacts. The removal treatments described herein include physicochemical, biological, and chemical treatment approaches, and advanced oxidation processes (AOPs). Also, alternative, green, and sustainable treatment options were discussed to shed insight into effective treatment technologies for herbicides. To conclude, this review serves as a stepping stone to a better environment with herbicides.

## Introduction

The widespread use of synthetic pesticides has numerous benefits for the global population, particularly when it comes to protecting crops from pests and disease. According to Tostado and Bollmohr [1], the top 10 pesticide using countries are China, the United States, Argentina, Thailand, Brazil, Italy, France, Canada, Japan, and India. With 407.8 thousand tonnes of pesticide used in 2020, the United States ranks first in pesticide use, followed by Brazil with 377.2 thousand tonnes. In total, 2.66 million metric tonnes of pesticide were consumed globally in 2020. Tostado and Bollmohr [1] reported that global pesticide use has doubled between 1990 and 2022 to 4 million tonnes total. With an annual growth rate of more than 4% since 2015, the size of global pesticide market grew to 84.5 billion US dollars in 2019, and the growth rate is likely to increase in the future. About 60% of the pesticides used today is herbicide. Furthermore, most large-scale crop production systems depend heavily on synthetic herbicide to control weeds [2].

Pesticide prevents the spread of diseases that can ruin entire crops and allows crops to grow and mature providing sustainable global food supply [3]. Pesticide is one of the emerging contaminants and herbicide is a type of pesticide designed to target and control unwanted plant growth [4,5]. These chemicals are used to kill or suppress weeds and unwanted vegetation in a wide variety of settings, such as agricultural fields, lawns, gardens, and industrial sites.

Herbicide is composed of tiny molecules (typically 500 MW) that primarily target the physiological processes of plants [2]. Herbicide works by interfering with the plant’s metabolic processes causing them to die or cease growing. Depending on the types and application methods, herbicide can be selective, targeting only certain plants, or nonselective, affecting all vegetation [6]. According to Nandula [7], herbicide quickly supplanted other weed management methods due to its superior effectiveness, cost-effectiveness, selectivity, and targeted weed control. For every conceivable cropping scheme, at least one herbicide has been approved for use.

Herbicide offers increased productivity, improved produce quality, decreased labor-intensive hand weeding, and decreased soil erosion and topsoil loss due to requiring less cultivation and tillage (enhanced by less fossil fuel use). While herbicide is effective in controlling weeds and unwanted vegetation, it has negative environmental and human health impacts. For example, there is a strong link between work exposure and disease, particularly cancer. Tin light of this, safety and health precautions must be taken when handling herbicides [8]. Certain herbicides can be vulnerable to volatilization, leaching, and runoff, which may cause them to accumulate in soils, water bodies, and tissue. Additionally, these substances could harm unintended organisms. The lengths of time herbicides persist in different environmental compartments vary significantly, ranging from those that break down quickly into harmless by-products, to those that persist in the environment [9]. Furthermore, extensive herbicides use can lead to plants developing resistance to them, either as a natural selection process or through intentional resistance in genetically modified organism (GMO) crops [3–5].

There are several herbicide types used for various treatments for promoting growth. According to the United States Environmental Protection Agency (U.S. EPA), the 10 most widely used herbicides on US agricultural land are glyphosate, imazethapyr, thifensulfuron as amino acid inhibitors; atrazine, cyanazine as photosynthesis inhibitors; 2,4-D, dicamba as synthetic auxin growth regulators; trifluralin, pendimethalin and metolachlor as cell division inhibitors. Glyphosate, atrazine, and 2,4-D are the leading herbicide applications. Other types of herbicides include urea, propachlor, metribuzin, fenuron, fluometuron and monuron, which are also identified as water pollutants [10].

Herbicide is applied by spraying onto foliage, soils, and aquatic systems. It can indirectly enter surface water through runoff or leachate, causing contamination and the biological impairments of water bodies and ecosystems [11]. Herbicide pollutes aquatic ecosystems in four ways, 1) by direct application to environmental waters; 2) by migration from crops and soil into the environment via runoff and spray drift; 3) through excessive use in agricultural practices; 4) through accumulation in aquatic environments [12]. Herbicide is chemically and photochemically stable in mild conditions making it difficult to be degraded by physicochemical and biological processes in wastewater treatment facilities [10]. It accumulates in soil and causes adverse effects on soil life for decades [1]. The heavy use of herbicide can expose non-target plants, animals, and humans with profound effects on ecosystem functions and microbial communities in the environment [13].

Moreover, herbicide accumulation transfers across species through the food chain and eventually reaches humans. Although precaution, awareness and enforcement have been implemented to minimize herbicide release, the residue persists in groundwater. Recently, trace herbicide was detected in drinking wells in the US [14], Canada, China [15,16], Japan [17], Brazil and Vietnam [18]. This contaminant is globally widespread and disrupts the ecosystem. More research and action are needed to address this issue and minimize the impact of herbicide on human health and the biosphere. Thus, the present review is aimed to summarize and discuss the classification of herbicides, their health effects, and various treatment strategies to abate their occurrence, as well as the challenges and future outlook toward a sustainable environment.

## Classification of herbicides

The first commercially used herbicide reported was 2,4-Dichlorophenoxyacetic acid, 2,4-D [19]. In practice, 2,4-D is a herbicide generally used in non-agricultural contexts, and in 2012, it was the fifth most widely used herbicide in the US agricultural sector [20]. The highest cause for crop loss is weeds, accounting for 34% of loss. This is followed by insects (18%) and pathogens (16%) [21]. Herbicide offers high crop management efficiency, and it reduces the need for labor and mechanical energy [21,22]. In the environment, herbicide undergoes degradation and migration. Degradation is initiated through microbes, chemical reaction, and light (photodegradation), and it generates various simple metabolites [22]. Sometimes metabolites are more potent, mobile, and toxic than the parent herbicide, and this can increase their environmental persistence (e.g. soil, water, and/or air). At huge plantation areas, herbicide spray droplets may recede, volatize, and drift from the site via air currents. These spray droplets can reach other soil surfaces binding with soil particles and potentially diffusing into deeper soil layers with greater permeability where they persist for considerable periods [22,23]. Herbicide residue also dissolves in eroding soil water ending as runoff that ultimately reaches water bodies. Forestry and silvicultural management practices, horticultural and agricultural operations, urban development and maintenance, and industries including herbicide manufacturing are all potential herbicide sources that engender surface water herbicide and resulting ecosystem impairments [24]. In forest management, herbicides are employed to get logged-out areas prepared for new planting. Among other things, lawns, parks, golf courses, and even streams are treated with herbicide. Herbicide application to streams is used to control aquatic weed growth.

Herbicide is classified depending on several aspects, such as time and method of application, selectivity, mode of mobility, mode of action, residual action in soil, chemical structure and formulation [25,26]. With respect to time of application, herbicide is categorized as pre-plant, pre-emergence, and post-emergence. The terms ‘pre-plant application’ is designated for volatile herbicides, such as fluchloralin and trifluralin. These volatile herbicides are used a day (or just) before crop sowing [27]. The terms ‘pre-emergence herbicide’ is designated for chemical agents applied to the soil one to two day after sowing to eliminate grassy (or broad leaf) weeds that compete with the crops during germination [26]. The common herbicides under this category include atrazine, alachlor, butachlor and metribuzin. ‘Post-emergence application herbicides’ refer to chemical agents such as 2,4-D, sulfosulfuron, and isoproturon, which are applied after the weeds and crop emerge. Herbicides for pre- and post- emergence are mostly selective. They are formulated to kill specific weeds without harming nontarget crops. By contrast, nonselective herbicides such as paraquat, diquat, picloram, amitrole, and glyphosate are occasionally used when the land needs to be completely cleaned from all vegetation or in cases where the weeds are located far from the crops. However, glyphosate becomes more selective when applied to crops that have been genetically modified to resist it [28,29].

The terms ‘application method’ are herbicides that are applied directly to soil or crop foliage to kill germinative weeds [26]. Spraying herbicide uniformly to surface or subsurface soil can control perennial weeds. Alternatively, fumigation and herbigation can be applied at soil sites. Fumigation method involves volatile chemical application in confined spaces, and herbigation is performed along irrigation systems. Application method involves the use of chemical agents such as methyl bromide, metham sodium, or chloropicrin in strawberry fields [30]. Herbicide application to foliage, on the other hand, is commonly used to control annual and perennial weeds and woody shrubs by blanket (or directed) spraying. ‘Blanket spray method’ involves uniform herbicide application without consideration to crop location (e.g. spraying of 2,4-D ethyl ester to the paddy field). Directed spraying involves specific application to weeds (e.g. *Cyperus difformis* and *Cyperus rotundus*) avoiding crops [31]. Protected spray method involves covering spray areas with polyethylene, and weed foliage spot treatment is typically implemented in small areas that are weed infested.

The classification of herbicide through its mode of mobility takes into account the routes it follows upon contact with weed. ‘Systemic herbicide’ refers to the mobility mode wherein absorbed herbicide is translocated into the weed’s xylem and phloem [26]. Another mobility mode is called ‘contact herbicide,’ wherein the herbicide is less (or not) mobile and kills weed upon direct contact. The mechanism by which herbicide kills weeds is referred to as the mode of action (MOA). Classification by the herbicide’s MOA is unstandardized, as researchers’ do not uniformly agree about classifications. Granting that, this review paper gathers the most common MOAs.

Generally, it is crucial to comprehend the MOA to choose the appropriate herbicides for a given crop, recognize the effects on the weeds, and plan an effective crop-management strategy [26]. If these elements are not in order, the weed can become dominant, resulting in ineffectiveness, and necessitating increased herbicide levels. Eventually, herbicide accumulates in the environment and requires treatment. Herbicide MOA can be divided into the several types as follows: photosynthesis, specific enzyme target, cell division disruption, seedling growth inhibitor, and synthetic auxins. MOAs involving photosynthesis involve inhibition in photosystem I and II (PS I and PS II) making the weed yellow, desiccated from the tips, edge, and between veins. Protoporphyrinogen oxidase (PPO) in foliar-applied herbicide inhibits the photosynthesis of weed, which engenders the rapid desiccation of green tissue when in contact [32]. Carotenoids are essential components of photosynthetic tissue and if the inhibition of carotenoid biosynthesis occurs, the shoots become bleached leading to cell death [33]. Glufosinate ammonium herbicide controls weed by inhibiting glutamine synthetase, which prevents the conversion of glutamate and ammonia. This inhibition causes ammonia accumulation in weeds, which damages cells and directly inhibits PS I and PS II interactions, resulting in a yellowing or bronzing effect on the shoots [34].

Inhibition of specific enzymes target enzymes such as acetyl coenzyme A carboxylase (ACCase), acetolactate synthase (ALS), and enolpyruvylshikimate-3-phosphate synthase (EPSP). The weeds stop making fatty acid (inhibition of ACCase) and amino acid (inhibition of ALS and EPSP synthase) during pre- and post-emergence applications. The growth of weed can stop within hours, stunting shoots and roots, yellowing the leaf and causing death within days or weeks [35,36]. In the southern United States, crop production glyphosate-resistant palmer amaranth *(Amaranthus palmeri)* is one of the most difficult weeds to control. Accordingly, the herbicides metolachlor has been extensively researched for its ability to restrict seedling growth by disrupting its cell division [37]. Meanwhile, the application of synthetic auxin herbicides such as 2,4-D during post-emergence has the effect of twisting weed stems and curling the leaf within hours [38]. Non-residual or zero persistence herbicides involve chemical agents that quickly breakdown leaving no residue in soil. Conversely, triazines and imidazolines are examples of residual herbicides. They are more resistant to degradation and continue to work effectively against weeds over an extended period. Although residual herbicides are useful for agricultural purposes, they pose a serious threat to ecosystems, communities, human health, and organisms because they remain present in residual amounts in soil, water, and nontarget plants [39].

**Figure 1.**
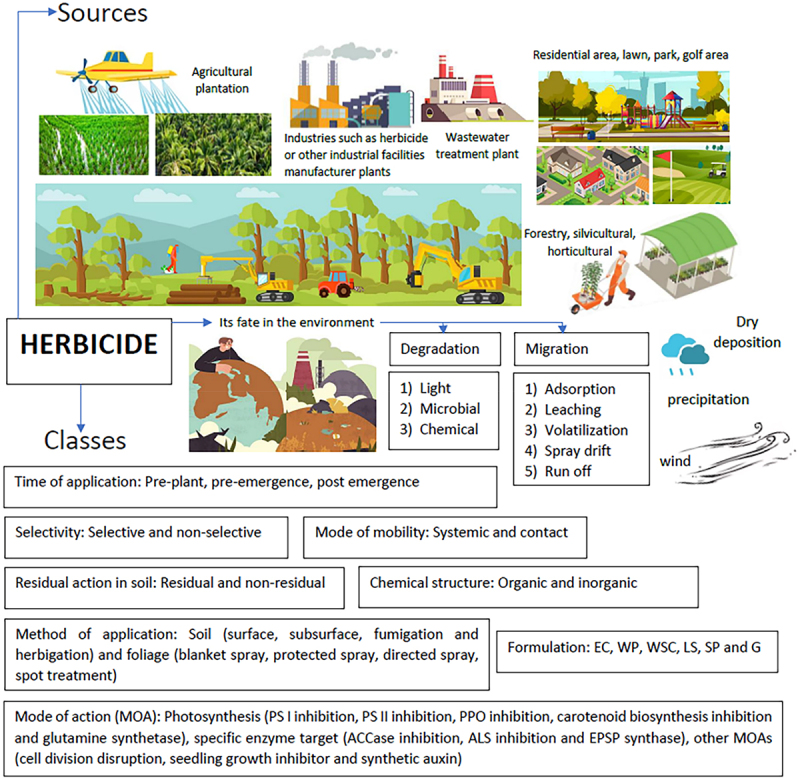
The summary of the environmental fate of herbicides, including their sources and classification.

Another herbicide classification is based on its chemical structure, which can be either inorganic or organic. Inorganic herbicide such as 2,4-D was the first herbicide used for weed control prior to the introduction of organic-based herbicides. The final herbicide classification is through its formulation. The six subcategories depend on whether the herbicide is in emulsifiable concentrate (EC), wettable powder (WP), water soluble concentrate (WSC), liquid suspension (LS), soluble powders (SP) or granule (G). The summary of the environmental fate of herbicides, including their sources and classification is shown in Figure 1.

## Health effects of herbicides

Hazardous pesticides are a global problem because of its tendency to bioaccumulate in human cell membranes and disrupt the body’s functioning system. Its widespread use has engendered worldwide fatalities and health complications due to occupational exposure and intentional poisoning [40]. Environmental contamination and general use have exposed humans to pesticides through dermal contact, inhalation, food and water consumption [41]. Its direct and indirect effects, however, are difficult to identify and costly [40,42]. According to the United States Environmental Protection Agency (U.S. EPA) [43,44], pesticide type, exposure duration, and the individual’s health status (e.g. nutritional deficiency and healthy/damaged skin) influence the health consequence’s severity. Children are more susceptible to pesticide’s adverse effects due to their smaller size (based on milligrams per kilogram of body weight), different metabolism, and still-developing organs [41].

In human body fat, pesticides can be metabolized, expelled, stored, or bioaccumulated [44]. Although concentrations may not necessarily exceed legally established ‘safe’ levels, these levels could underestimate actual health risks, as real-world exposure may involve concurrent subjection to multiple synergistically interacting chemical agents [44]. The numerous adverse health effects associated with chemical pesticide exposure include toxicity, carcinogenicity, dermatological, gastrointestinal, neurological, respiratory, reproductive, and endocrine effects [43–46]. The effects can be further specified into short- and long-term effects. Short-term exposure can negatively affect the liver, kidneys, blood, lungs, neurological system, immunological system, and digestive system [41].

**Figure 2.**
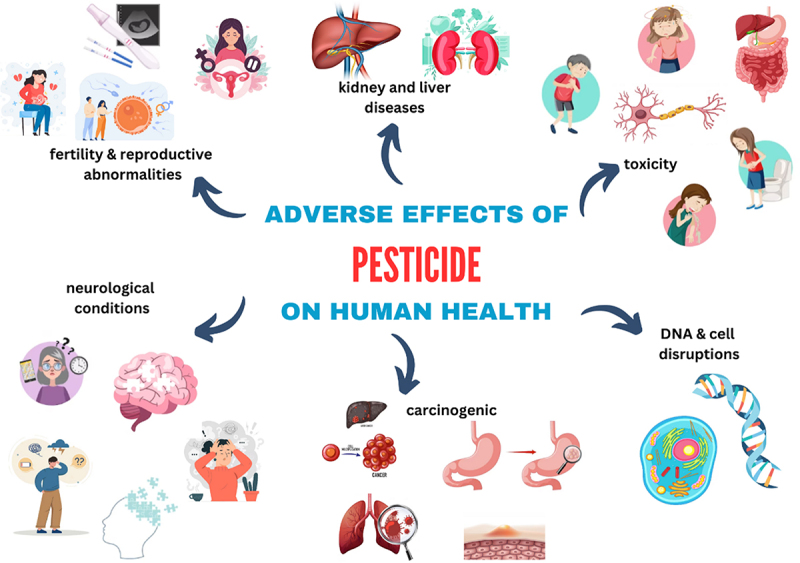
Adverse effects of pesticides on human health as different living systems.

According to Sameeha [47], short-term exposure commonly results from improper application and handling practices leading to direct contact with harmful chemical components. The short-term effects occurring from short-term exposure include skin rashes, nausea, diarrhea, stomach-ache, pain, and blisters caused by the formation of fluid-filled gaps between skin layers. Additionally, exposed individuals could experience dizziness, stinging eyes, and blindness if harmful chemicals came into direct contact with the eyes. Furthermore, inappropriate handling and unintentional contact could cause excessive salivation and mouth burns.Herbicide exposure could also result in long-term health effects, including DNA abnormalities leading to mutations. Herbicide produces reactive oxygen species (ROS) that are responsible for the oxidation of proteins, nucleic acids, lipids, and other cellular components, resulting in DNA damage. Eventually, cell damage and death inhibit numerous enzymes and cellular cycles. Other long-term effects include kidney damage, cancer and Parkinson's disease, which are attributable to central nervous system degenerative diseases. Additionally, birth complications and developmental abnormalities can contribute to the development of infants with developmental defects. These include behavioral changes, missed developmental milestones, learning disabilities, attention deficit hyperactivity disorder (ADHD), and emotional disorders. Excessive levels of occupational, unintentional, or intentional pesticide exposure can lead to hospitalization and death [47]. Table 1 summarizes the health effects attributed to pesticides. The adverse effects of pesticides on human health are also presented in Figure 2.

**Table 1.** Health effects based on exposure to pesticides.

Short-term exposure	Skin rashes
	Skin blisters
	Nausea
	Diarrhoea
	Stomach-ache and pain
	Dizziness
	Stinging eyes
	Eye irritation
	Permanent failure of eyesight and blindness
Long-term exposure	DNA abnormalities and mutations
	Cell damage and death
	Kidney damage
	Cancer
	Parkinsonism
	Birth problems during pregnancy
	Developmental abnormalities and defects
	Excessive salivation and mouth burn

## Health effect 1 - toxicity

All pesticides must be biologically active or toxic to be effective against pests. Pesticide can be dangerous for people, animals, living organisms, and the biosphere since it is poisonous [48]. Acute and chronic toxicities are observed with herbicide exposure. The formulation of pesticide can have a big impact on both toxicity and exposure [41]. Numerous herbicides have mild to moderate acute toxicity [42]. Acute toxicity refers to a chemical’s capacity to harm a person from a single exposure, often of short duration, and it varies greatly depending on the type of pesticide. It is assessed by analyzing dermal toxicity, inhalation toxicity, and oral toxicity in test animals, in addition to its level of eye and skin irritation. Common short duration exposure side effects also include reduced eyesight, headaches, salivation, diarrhea, nausea, vomiting, wheezing, coma, and death. Moderate pesticide poisoning causes intrinsic asthma, bronchitis, and gastroenteritis [41]. Chronic pesticide exposure negatively impacts the skin, eyes, brain system, cardiovascular system, gastrointestinal tract, liver, kidneys, and reproductive system [42,48]. In addition, exposure through water ingestion can mimic human hormones, which can lower immunity, disrupt the endocrine system, cause hormone imbalance, increase cancer risk, and reduce intelligence, especially in developmentally maturing children [40,48].

## Health effect 2 - DNA and cell disruptions

Several reports have shown significant cytogenetic damage due to exposure with various types of pesticides [49]. A wide range of chemical agents used to formulate pesticides contributes to DNA damage [50] because of the alteration in the chemical structure and sequence of the DNA [51]. Pesticides are reported to induce DNA adducts and cause DNA single or double strand breaks. Also, the produced DNA lesions hinder the genome replication and transcription, which could subsequently result in mutations or wide-scale genome aberrations, thus threatening the cell or organism viability [52].

According to several studies, glyphosate-based herbicide has been found to cause DNA damage in the liver and kidneys of mice [42]. It has been reported that 2,4-D and dicamba herbicides can cause DNA damage in Chinese hamster ovary (CHO) cells and human lymphocytes at the chromosomal and DNA levels. In addition, both herbicides can cause Sister Chromatid Exchanges (SCEs) in mammalian cells with clastogenic activity. The chromosomal aberrations in human lymphocytes may also occur among pesticide-exposed workers [42].

## Health effect 3 - carcinogenic

In 2015, the International Agency for Research on Cancer (IARC) of the World Health Organization concluded that glyphosate-based herbicide is probably carcinogenic to humans [43]. Also, an Agricultural Health Study produced evidence that increasing lifetime exposure to a specific pesticide increased the incidence of childhood cancer, prostate cancer, lung cancer, colon cancer, pancreatic cancer, bladder cancer, leukemia, and multiple myeloma [41]. Another report revealed that frequent exposure to chlorinated pesticide increased the risk of prostate cancer among farmers [40,42]. In addition, clinical studies conducted by Cancer Risk Research revealed that glyphosate users had a higher incidence of non-Hodgkin lymphoma, a rare form of cancer, relative to non-glyphosate users [42].

## Health effect 4 - fertility and reproductive problems

Pesticides causing endocrine disruption, decreased sperm count, or increased sperm abnormalities have been identified [42]. A few studies have assessed whether pesticide exposure diminishes sperm quality, decreases sperm count, and alters sperm morphology [40]. The rabbit tests with glyphosate have demonstrated dose-dependent negative effects on sperm quality [42]. Miscarriage, infertility and malformation, dermatological and respiratory illnesses are also associated with pesticide residue in agriculture produce and feed [53].

## Health effect 5 - liver and kidney-related diseases & neurological conditions

Nutritional experiments with glyphosate lifetime have demonstrated a reduction in body mass, adverse effects on liver and kidneys, and damage to the crystalline lens of the eye [42]. In some localities with heavy glyphosate use and ‘hard’ water, male agricultural workers presented a higher prevalence of chronic kidney disease [54]. Increased levels of glyphosate use have been linked to several mental illnesses, including Alzheimer’s, Parkinson’s, Attention-Deficit/Hyperactivity Disorder (ADHD), and autism [44,53]. Van Bruggen [53] found that glyphosate exposure causes DNA damage in leucocytes at moderate to high concentrations and decreases DNA methylation in in vitro tests on human peripheral blood cells. The delicate balance between cell proliferation and programmed cell death can thus be disturbed by glyphosate, which interferes with neurotransmission. Several correlational and cellular studies have shown that persistent low exposure to glyphosate can affect the activity of acetyl cholinesterase enzyme at the organismal level resulting in serious neurological disorders, as nerve impulses are not switched off.

## Current removal technologies for removal of herbicides

### Physico-chemical treatment

Adsorption is a popular method for removing herbicides from water due to its cost-effectiveness, easy to use and resistance to harmful substances. It is the process by which a gaseous or liquid part sticks to a solid surface like activated carbon, zeolite, or clay. These solid surfaces are called ‘adsorbents.’ The effectiveness of the process depends on operational factors, such as adsorbent dosage, pH, contact time, temperature, and initial adsorbate concentration. Recently, McGinley et al. [55] have reported on the use of many reusable media as adsorbents for herbicides such as granular activated carbon, peat fiber, bottom ash, fly ash, blast slag, Phoslock©, and zeolite. Of these, granular activated carbon obtained the best herbicide removal with more than 95% removal capacity. The increasing demand and widespread use of AC have prompted continuous studies to produce AC either with better herbicide removal capacity, or cheaper production cost. Agriculture waste and natural biomass-based materials such as coconut wastes [56], orange pulp [57], chitosan [58] lignin [59] and foxtail palm fruit [60] have been studied for herbicides removal.

Another potential adsorbent is biochar, which has a porous structure rich in carbon content. This type of charcoal is produced through the pyrolysis process. Ma et al. [61], removed 2,4-dichlorophenoxyacetic acid (2,4-D) from aqueous solution by utilizing H_3_PO_4_^−^activated spent coffee ground (SCG) biochar. The optimal removal was achieved at pH 2 with an adsorbent dosage of 0.75 gL^−1^. The maximum adsorption capacity was 323.76 mgg^−1^, which is among the top recorded for 2,4-D adsorptive studies. Previously, Essandoh et al. [62] reported the removal of 2,4-D and 2-methyl-4-chlorophenoxyacetic acid (MCPA) from simulated wastewater using switchgrass *(Panicum virgatum)* biochar. Switchgrass, a perennial C4 grass, has been gaining popularity for large-scale cultivation due to its high biomass yield, high bioenergy potential, ability to improve soil quality, and ability to reduced fertilizer and water demands. Compared with commercial activated carbon, which has a significantly higher surface area, switchgrass biochar can adsorb both pollutants remarkably well, with maximum adsorption capacities of 134 mgg^−1^ and 50 mgg^−1^ for 2,4-D and MCPA, respectively, at pH 2. Furthermore, it can withstand a diverse variety of pH levels.

Clays have also been studied as low-cost adsorbents of herbicides. Calisto et al. [63] used a Layered Double Hydroxide (LDH) to remove 2,4-D from contaminated water. This finding is interesting, because even though LDH is frequently used as electrodes in supercapacitors, the [Co – Al–Cl] LDH synthesized by the co-precipitation method showed mesoporosity, a high specific surface area and an excellent adsorption affinity for 2,4-D. It was reported that a maximum removal of 2,4-D was achieved after 60 minutes of contact time, which is fast relative to other types of adsorbents.

Recently, Wu et al. [64] used an inverse suspension polymerization method to make a bifunctional polyethyleneimine-grafted lignin microsphere (PLM). These microspheres effectively bind with 2,4-D molecules in a variety of pH ranges (pH 4 to 10) under mild conditions due to their high porosity, availability of functional groups, and suitable surface charges. The highest 2,4-D adsorption capacity (909.09 mgg^−1^) was appreciably more significant relative to other adsorbent materials reported in previous studies. As lignin has a limited specific surface area and no active sites reducing its adsorbate affinity, the use of PLM as an adsorbent to remove herbicides is beneficial [65]. However, the distinct lignin macromolecule structure makes it possible to modify its properties by grafting, crosslinking, polymerizing, or substituting it with other active moieties to engender the performance required for a specific purpose [59,66].

Many studies also showed that composite adsorbents offer greater cation exchange capabilities, improved mechanical strength, specific pore size, and higher surface area. In addition, these materials can be recycled. Sugarcane bagasse (SB) composite with polyaniline (PAN), polypyrrole (Ppy) and sodium alginate (NaA) were used as an adsorbent for 2,4-D removal by varying the pH, temperature, contact time and composite dose [67]. All of the bio-adsorbents could remove 2,4-D at the ideal condition of pH 3, a composite dose of 0.05 g/50 mL, 29.9°C and 90 minutes of contact time.

The emergence of nanotechnology has expanded nanocomposite material use such as metal organic frameworks (MOFs), carbon nanotubes (CNT), and graphene oxides (GO) to enhance the adsorption process. One of the most utilized two-dimensional materials is graphene. Relative to graphene, GO possesses more surface oxygen functional groups, such as hydroxyl, carboxyl, and epoxy. These hydrophilic groups make GO an ideal substrate for esterification and amination derivative processes that provide novel characteristics. GO or modified GO removes harmful and dangerous pollutants from contaminated surroundings [68]. By functionalizing GO with a polymer to improve its adsorption performance, GO dispersion can be enhanced [69,70]. Li et al. [71] have successfully composited poly N-isopropylacrylamide (PNIPAM) with GO through surface-initiated atom-transfer radical polymerization for sulfonylurea herbicides (SUH) removal in aqueous solution. This grafted composite was used successfully to remove SUH after one minute of contact time. For the removal of 2,4-D and glyphosate from water, magnetic nanoparticles coated with activated silica using rice husk ash modified by the 3-Mercaptopropyltrimethoxysilane (Thiol) functional group have also been reported [72]. Utilizing magnetic nanoparticles has enhanced the removal of 2,4-D and glyphosate. Indeed, even after five cycles, the adsorption capacity did not significantly decrease, suggesting its stability and reusability potential.

Another promising method for removing herbicides from water sources is membrane separation, including nanofiltration (NF), reverse osmosis (RO), and forward osmosis (FO). Atrazine (ATZ) removal from the water was studied by Chandra et al. [73] using polyamide microfiltration (MF) membranes with functional layers of chitosan/polystyrene sulfonate (CHI/PSS). The removal percentage of ATZ increased from 56% to 99% when the bilayer was increased from 5 to 9 layers, respectively. Increased layers block more herbicide solutes from passing through the membrane. In addition, the solute has an increased tendency to be absorbed by the expanding number of interactive sites, contributing to increased efficiency. Recently, nanoparticle incorporation into membrane technology has garnered considerable interest. This is because adding nanoparticles improve the membrane’s separation performance by increasing membrane surface area. Moreover, the addition of nanoparticles changes the membrane’s surface characteristics. For example, it makes the membrane’s surface more hydrophilic and lowers membrane fouling. Furthermore, it strengthens the membrane’s mechanical properties, extends its lifespan, and prevents membrane damage. One example is the use of synthesized zinc nanoparticles (ZnNPs) in cellulose acetate to remove organic herbicides i.e. metolachlor and acetochlor [74]. It has been reported that adding 0.1 mg ZnNPs per 100 mL of 0.5 gL^−1^ herbicide successfully removed 99% of metolachlor and 61% of acetochlor. Additionally, incorporating ZnNPs into a polymeric membrane increases its lifespan (activity and reusability) and minimizes environmental concerns of the potential release of NP into the environment.

In a different study, a metal organic framework (MOF) mixed matrix membrane (MMM) was prepared by combining the MOF material with polymer [75]. This is recommended because of its substantial porous structure, high specific surface area, expandable pore diameter, variety of structure and function [76], unsaturated metal sites, and excellent biocompatibility [77]. In order to treat sulfonylurea herbicides (SUHs) in an aqueous environment, porous materials, such as the MIL-53 type MMM, were combined with poly (vinylidene fluoride) (PVDF) polymer. These materials demonstrated a high herbicide removal capacity of up to 25 cycles, indicating their viability in practical applications [75].

Several studies examined the efficacy of liquid membranes in removing herbicides from aqueous solutions. One membrane form is a liquid membrane, which uses a liquid phase to selectively extract and transports target molecules or ions from a mixture. Examples of liquid membranes include polymer inclusion membranes (PIM), supported liquid membranes (SLM), and bulk liquid membranes (BLM). Mwakalesi and Potter [78] described the use of a cellulose triacetate-based PIM containing trioctylmethylammonium chloride (Aliquat 336) and 2–nitrophenyl octyl ether (NPOE) for picloram removal from aqueous solutions. With an optimized receiving solution of 0.25 M NaCl, picloram obtained a maximum flow of 294 × 10^−8^ molm^−2^s^−1^ and a transfer efficiency of 95%. The PIM showed the capacity to extract picloram at the highest concentration of 500 gL^−1^ from a complicated natural water matrix using a passive sampling instrument. However, other anions in natural water can also pair up with Aliquat 336 to make ion-pairs, which could reduce its effectiveness in removing picloram. In recent work, 1-butyl-3-methylimidazolium hexafluorophosphate [BMIM]^+^[PF_6_]^−^ as an extractant was immobilized using the vacuum method in the PVDF membrane for 2,4-D removal [79]. An effective removal was observed when the conditions process was 50 mgL^−1^ phenol, 0.1 M NaOH stripping agent, pH 4 and 400 rpm rotation.

In summary, there are various methods available to mitigate the environmental and health impacts of herbicides in water. Treatment effectiveness may depend on the type of herbicides, strength of the water source being treated, and the overall unit operation involved in the treatment process.

## Biological treatments

Herbicide degradation involves chemical breakdown into less harmful or nontoxic substances. Biological degradation occurs when soil or water microorganisms use herbicide as a food or energy source. Glyphosate is among the most extensively utilized herbicides worldwide. It is a nonselective, systemic herbicide that inhibits 5-enolpyruvylshikimate-3-phosphate (EPSP) synthase. Borella et al. [80] revealed that a bacterial consortium consisting of *Pseudomonas stutzeri*, *Comamonas odontotermitis*, and *Sinomonas atrocyanea* reduced glyphosate concentrations from 53% to 79% at 5 to 50 mgL^−1^ of glyphosate in both synthetic and real wastewater. Notably, glyphosate was successfully degraded without aminomethylphosphonic acid formation (AMPA).

Zhang et al. [81] pointed out another environmentally favorable biological method used for removing glyphosate residue from soil. It was discovered that *Chryseobacterium* sp. (a novel bacterial strain that effectively breaks down glyphosate and its primary metabolite AMPA) strain Y16C only took 96 hours to completely break down glyphosate at a 400 mgL^−1^ dosage. In addition, Rossi et al. [82] successfully isolated four bacterial strains that are taxonomically affiliated to CNII15, to wit, *Acidovorax* sp. CNI26, *Agrobacterium tumefaciens* CNI28, *Novosphingobium* sp. CNI35 and *Ochrobactrum pituitosum* CNI52. Glyphosate and AMPA were degraded completely by all strains in 125–400 hours and 30–120 hours, respectively.

In a study by Masotti et al. [83] *Achromobacter, Agrobacterium*, and *Ochrobactrum* were able to grow and consume glyphosate-based herbicides (GBH), glyphosate, or aminomethylphosphonic acid (AMPA) with degradation percentages ranging from 41–56% after 96 hours. Firdous et al. [84] isolated the novel bacterial strain *C. odontotermitis* P2 from glyphosate-contaminated field soil in Australia and it degraded 0.54 gL^−1^ glyphosate completely in 104 hours, with a maximum degradation rate of 90%. In another study, three bacterial strains with remarkable glyphosate-degrading abilities were isolated by Singh et al. [85] from agricultural soils. *Streptomyces* sp., *Bacillus subtilis*, and *Rhizobium leguminosarum* utilized 85–90% of the glyphosate in the thirty-day experiment, with half-lives ranging from 8.36 to 9.12 days.

Other microorganisms were also involved in herbicide biodegradation. *Arthrobacter* sp. has been reported to degrade trifluralin [86], atrazine [87], and isoproturon [88] into nontoxic compounds. In addition, *Bacillus subtilis* exhibits a high capability to degrade pendimethalin [89] and nicosulfuron [90]. Recent research conducted by Liu et al. [91] revealed the mechanism of *Rhodococcus* sp. B2 in the bioremediation of pretilachlor-contaminated soil. Moreover, the white-rot fungus *Rigidoporus* sp. can degrade 2,4-D and 2,4,5-trichlorophenoxyacetic acid (2,4,5-T) by producing laccase and cytochromes P450-type for the breakdown of the herbicides [92].

Overall, microbial herbicide degradation is a promising method for reducing herbicide levels, but the process is much slower (taking weeks or months to complete) and it is directly influenced by factors such as pH temperature, moisture content and other chemicals. During this time, the herbicides can still be active and harmful to the environment.

## Chemical treatments

Hydrogen peroxide treatment can be enhanced to an advanced oxidation processes (AOPs) by incorporating ultraviolet (UV). This can further be combined with catalyst [93]. Photolysis of water by UV light (reaction 1) and ozone decomposition in water generate hydroxyl radicals (•OH) [94–97]. Application of ozone based AOPs can further accelerate •OH and lead to the formation of hydroperoxyl radical ($HO_{2}^{∙})$as shown in following reaction (reaction 2 to 4). Adding H_2_O_2_ to the herbicide treatment correspondingly produced the oxygen to the solution. Its presence eventually leads to the formation of superoxide radicals’ anions when reacting with the electron (reaction 5), thus enhancing the herbicide degradation process [98,99].

(1) $$H_{2}O\overset{hv}{\to }∙OH+∙H$$

(2) $$O_{3}+H_{2}O\overset{hv}{\to }H_{2}O_{2}+O_{2}$$

(3) $$H_{2}O_{2}\overset{hv}{\to }2∙OH$$

(4) $$∙OH+H_{2}O_{2}\to HO_{2}^{∙}+H_{2}O$$

(5) $$O_{2}+e^{-}\to O_{2}^{∙-}$$

Ikehata and El-Din [100] reported that ozone based AOPs such as ozone/hydrogen peroxide, ozone/ultraviolet irradiation, and ozone/hydrogen peroxide/ultraviolet irradiation, possess a high potential for degrading and detoxifying four major groups of herbicides, namely, aniline-based compounds, pyridines and pyrimidines, triazines, and substituted urea relative to ozone alone. Other inorganic oxidizing agents such as persulfate and peroxymonosulfate can also be used to degrade chloroacetanilide group herbicides such as alachlor and butachlor. Although the degradation process was expected to accelerate in the presence of hydroxyl radical, the alachlor degradation kinetics of various UV-based AOPs were highly dependent on the pH. Indeed, the UV/$S_{2}O_{8}^{2-}$ and UV/H_2_O_2_ processes were most effective under acidic conditions (pH 5), and the UV/${HSO}_{5}^{-}$ process demonstrated the highest alachlor degradation efficacy under basic conditions i.e. pH > 8 [101]. A study by Wen et al. [102], however, revealed that sulfate radical $\left({SO}_{4}^{∙-}\right)$ of peroxymonosulfate accounted for 75–78% of atrazine degradation rather than the direct oxidation by peroxymonosulfate, which only achieved 22–25%. A positive effect on the COD removal efficiency of 2,4-D was also obtained in the presence of hydrogen peroxide and persulfate anions [103]. On the other hand, the formation of formamides intermediate occurred during oxidation of chlorophenylureas by the O_3_/H_2_O_2_ [104].

Tran et al. [105] conducted a study on glyphosate herbicide degradation by an electro-Fenton process utilizing a carbon felt cathode. It was found that the maximum removal percentage of 0.1 mM glyphosate was 91.91% with an applied current density of 10 mAcm^−2^, under the conditions of pH 3, 0.1 mM Fe^2+^, and 0.05 M Na_2_SO_4_, after 40-minute of treatment. Total phosphorus (TP) removal was used to track the breakdown of glyphosate (reaction 6), with higher TP removal indicating greater glyphosate removal [90]. Using a Fenton-like system as a source of hydroxyl free radicals and Fe^2+^ as a catalyst to remove glyphosate from aqueous solutions, 99.67% of TP was removed in 30 minutes [106]. Meanwhile, 5 mgL^−1^ of 2,4-D was completely removed in 90 minutes using FeO@Fe_3_O_4_ nanoparticles combined with H_2_O_2_ to form a heterogeneous Fenton-like system. The dispersibility and stability of FeO@Fe_3_O_4_ nanoparticles was better than single nano zero-valent iron (nZVI) [107].

(6) $$C_{3}H_{8}NO_{5}P+OH\to CO_{2}+H_{2}O+NO_{3}^{-}+NH_{4}^{+}+PO_{4}^{3-}$$

Complete degradation of atrazine is achieved using TiO under 306 nm ultraviolet irradiation [108]. Another study was used Ag_3_PO_4_/TiO_2_ nanoparticles photocatalyst under visible light to degrade 2,4-D herbicide. The best result (98.4% removal efficiency) was obtained with a pH of 3, a catalyst dose of 1 gL^−1^, a contact period of 60 minutes and an initial 2,4-D concentration of 10 mgL^−1^. The results show that after five cycles of the procedure, the 2,4-D removal efficiency has decreased to 96.1% from 98.4% [109]. In another study, two different photocatalysts which were TiO_2_/Al_2_O_3_/carbon nanotube nanocomposite and TiO_2_/Al_2_O_3_/graphene were utilized for photocatalytic degradation of metamifop where photodegradation percentage were 95% and above in the presence of UV light and 2 Lmin^−1^ of air [110,111]. Khan et al. [112] found that TiO_2_ can efficiently catalyzed the degradation and mineralization of herbicide derivatives chloridazon, and metribuzin in the presence of UV light. In another chemical treatment approach, acid-treated graphene (G-COOH) catalyst was combined with ozone-activated peroxymonosulfate, while in a different investigation, G-C_3_N_4_ co-doped with phosphorus and kalium was used [113]. Atrazine was the subject of all mentioned treatments, which resulted in decreased atrazine toxicity and improved catalyst performance. It can be concluded that it is crucial to select the best degradation conditions in order to achieve high levels of mineralization and degradation, which are necessary for any practical use of photocatalytic oxidation processes.

Herbicides can be effectively removed from water using physico-chemical, biological, or chemical treatment methods, but in order to achieve mineralization, their implementation demands careful consideration of a number of variables, including the type of herbicides and their chemical structure, the type of treatment used, the ideal environmental conditions, the nature of the water matrix, the accessibility of particular equipment and resources, operational costs, and potential byproducts produced. The current technologies for herbicide removal based on physicochemical, biological, and chemical treatment are summarized in Table 2.

**Table 2.** Summary of some currently available herbicide removal technologies.

Physico-chemical/Biological/Chemical Treatments		Herbicides	Optimum conditions	q (mg/g or mmol/g) or R%	Reference
Type of adsorbents	Foxtail palm fruit activated carbon	Metamifop	10 ppm metamifop	86.65%	[60]
	H3PO4-activated spent coffee ground (SCG) biochar	2,4-D	pH 2 with adsorbent dosage of 0.75 gL^−1^.	323.76 mgg^−1^	[61]
	Bifunctional porous polyethyleneimine-grafted lignin microspheres	2,4-D	pH 4–10 removal efficiency decreased by 8% after 5 cycles)	909.09 mgg^−1^ at 45°C	[64]
	Thiol modified the magnetic mesoporous silica (magMCM-41) nanoparticles using extracted silica from rice husk ash	2,4-D glyphosate	herbicides concentration 20 mgL^−1^ adsorbent dose 0.2 gL^−1^ contact time 30 min (2,4-D) and 60 min for glyphosate. pH 6 for 2,4-D pH 5 for glyphosate	2,4-D − 83.44% Glyphosate − 79.38%	[72]
Type of microbes	Consortium activity of *Pseudomonas stutzeri, Comamonas odontotermitis*, and *Sinomonas atrocyanea*	Glyphosate	Glyphosate concentration: 5 to 50 mgL^−1^ No aminomethylphosphonic acid formation (AMPA).	53% to 79%	[80]
	Chryseobacterium sp. Y16C	Glyphosate	96 hours contact time 400 mgL^−1^ glyphosate concentration	Complete degradation	[81]
	Randomly methylated cyclodextrin + A. aurescens CTFL7	Trifluralin	19 days contact time 10 mgL^−1^ trifluralin concentration	88%	[86]
Type of Advanced Oxidation Processes (AOPs)	UV/H_2_O_2_ UV/S_2_O_8_^2-^ UV/HSO_5_^−^	Alachlor	UVC lamp, [S_2_O_8_^2-^]_0_ = [H_2_O_2_] = 0.3mM, pH = 5.0 UVC lamp, [HSO_5_^−^]_0_ = 0.3mM, pH = 9.5	Above 95.0%	[101]
	Fe^0^@Fe_3_O_4_/UV	2,4-D	[2,4-D]_0_ = 5.0 mgL^−1^, [Fe0@Fe3O4]_0_ = 0.5 gL^−1^, H_2_O_2_ = 1 mM, pH = 5.0 ± 0.2, T = 30°C	Completely removed in 90 min; nearly 66% of them could be mineralized.	[107]
	TiO_2_/Al_2_O_3_/carbon nanotube	Metamifop	20 mg of TiO2/Al_2_O_3_/CNT photocatalyst, [metamifop] = 10 mgL^−1^, air flow rate = 2 Lmin^−1^, 3 hours UV irradiation	95.0%	[111]
	P, K-doped g-C_3_N_4_ with cyano group and nitrogen vacancies (PKCN)	Atrazine	40 mg PKCN, [atrazine]_0_ = 10 mgL^−1^, Xe lamp of 300 W visible light (420 nm filter) for 60 minutes, pH = 5.0,	95.0%	[113]

## Alternative, green, and sustainable treatment technologies

The ever-increasing demand for chemical-based herbicide is due to its effectiveness to maximize crop productivity by controlling the growth of weeds that compete with crops for light, water, and soil nutrients [114]. The synthetic organics used in herbicides are known to be highly bio-recalcitrant and chemically stable in the environment [10]. Their mobility in soil is attributed to leaching and runoff that later contributes to agricultural non-point source pollution i.e. one of the major causes of reduced surface water quality [115]. With advances in research and development, the field is moving toward alternative technologies, as they are effective, green, and sustainable in addressing modern-day wastewater treatment [10].

At the top of the waste management agenda, simply avoiding chemical herbicide use is best, as no treatment would be necessary. Nevertheless, alternatives are available for example, combating weeds has been successfully done using natural extracts from, among other things, Nerium and olive [114]. This approach is based on the allelopathy effect, whereby plant extract biochemicals interrupt weed growth and survival. The plant extract is organic, biodegradable, eco-friendly, and pollution-free. Nonetheless, its use was examined on a small scale and the ecological and physiological properties of the extracts are not currently understood in the context of large-scale applications. Averting the arrival of chemical herbicide from water sources is another sustainable strategy to reduce the need for conventional remediation practices [115]. This can be done through mineralization using the microbial metabolism process, in which soil microorganisms consume herbicide compounds as a carbon source and turns them into NH_3_, H_2_O, and CO_2_ via photolysis and hydrolysis in the presence of enzymes (e.g. peroxidase, polyphenol oxidase, invertase, etc.) [116]. This in-situ-based treatment, however, typically suffers from a low biodegradation rate, and its effectiveness varies depending on microbe types and herbicide complexity [116,117]. Nevertheless, there is still a high probability of these chemicals finding their way into water bodies posing a threat to the aquatic ecosystem [96].

In wastewater and drinking water treatment facilities, some of the common facilities available for herbicide removal include UV photolysis, chemical oxidation, activated carbon adsorption, and microbial degradation with varying degrees of performance [116]. In the quest for high removal efficiency and sustainability, there have been several recent attempts to integrate conventional physical-chemical-biological treatment with the latest research findings [10,118]. For example, ‘nano-bioremediation’ is a term describing a hybrid process that combines bioremediation with nanotechnology through the immobilization of microorganisms onto nanoparticles. Nanomaterials, such as TiO_2_ and ZnO, have unique properties such as small size and high surface area to increase the interaction probabilities with target pollutants. By reaping the benefits of both moieties, the novel composite accelerates degradation and provides protection for the microbes to inhibit their self-degradation and deactivation thereby improving its reusability for increased reaction cycles [117,119]. Nevertheless, there are several challenges facing nano-bioremediation. These include the incompatibility of microorganisms, poor recyclability, and risks of nanomaterials in the environment [96]. Hitherto, electrochemical advanced oxidation [10] and photocatalysis [120] are among the new technologies that are widely studied in herbicide removal from water. Electrochemical advanced oxidation relies on electrochemically produced hydroxyl radical to mineralize herbicide molecules, while photocatalysis relies on light sources to generate electron-hole pairs in photocatalysts to excite degradation reactions. Novel photocatalysts have been developed in the aim to enhance photocatalytic efficiency via heterojunction formation, which encourages charge transfer between the two metals, thus suppressing the electron-hole recombination produced by photon absorption. This could be achieved by dipping in precious metal co-catalysts (e.g. platinum, palladium, silver, etc.) in semiconductor base photocatalysts (e.g. TiO_2_ and BiFeO_3_) [120]. Obviously, these techniques are primarily aimed at eliminating herbicides from water in a shorter period with little consideration regarding sustainability.

Developing functionalized biosorbents for efficient herbicide removal has become a research trend. Functionalized biosorbents are created by systematically engulfing herbicide molecules through a process called adsorption. The impetus for this research is to transcend the drawbacks linked to commercial activated carbons, such as high cost, poor removal performance, and high post-processing cost for reactivation or incineration. The strategy is based on the alteration of surface chemistry by introducing specific functional groups that could entrap herbicide molecules through electrostatic attraction, hydrogen bonding, complex formation and delocalized π–π interaction, on top of weak van der Waals’ forces and pore filling [118,121]. Aziz et al. [121] reported the preparation of polyaniline-functionalized biosorbent from *Brachychiton populneus* shell using aniline monomer and ammonium persulfate. Although the preparation features the use of abundantly available biomass, sustainable aspects are often overlooked in the use of chemicals during synthesis and reactivation. Moreover, most studies are conducted at bench (*vs*. industrial) scale. Thus, the performance has not been verified in the case where mass production cost is at stake. Table 3 summarizes the advantages and limitations for some of the available alternative treatment technologies for herbicides.

**Table 3.** Summary of the advantages and limitations for some of the alternative treatment technologies for herbicides.

Technique	Brief description	Advantages	Limitations	‘Green’ rating (1–5)	References
Herbicide substitution via allelopathy effect	Use plant extracts (Nerium, olives, etc.) as biological control of weeds	Natural resource Eco-friendly Pollution-free	Unknown ecological and physiological properties Limited to small scale	5	[114]
Nano bioremediation	Mineralization of herbicide compounds by microorganisms (e.g. F. mosseae, Pseudomonas strains) through hybrid nanomaterial-microbial remediation technique	Eco-friendly Low energy consumption Low operating cost Large-scale application	Slow rate of biodegradation Nanomaterials may bring negative effects to the environment and microbial activity. Poor recyclability, loss of microorganisms and nanomaterials High post-processing cost (incineration)	4	[96]
Electrochemical advanced oxidation	Herbicide molecules are broken by electrochemically produced reactive oxygen (hydroxyl radical) at the electrode surface (e.g. anodic oxidation, electro-Fenton, photoelectrocatalysis, etc.)	High efficiency Easy operation Harmless end products Able to mineralize less biodegradable atrazine	Energy-intensive process powered by electricity (high operating cost) Recalcitrant, toxic by-products/intermediates Limited to synthetic herbicide in small bench-scale	3	[10]
Functionalized biosorbent	The surface chemistry of biosorbent derived from biomass is altered by specific functional groups to entrap herbicide molecules through electrostatic attraction, complex formation, etc.	Natural resource High adsorption capacity Recyclability of material	Use of chemicals during synthesis and reactivation Limited to bench-scale Performance varies depending on the types of herbicides.	4	[121]
Advanced photocatalyst	Nanomaterial that absorbs light (photocatalysis process) to generate electron-hole pairs that promote redox (degradation) reactions of herbicide molecules into harmless compounds	High degradation performance Recyclability of material	Low quantum yield Very specific light source Toxic intermediates Leached nanomaterial may bring negative implications to environmental and public health	3	[120]

## Conclusion and future outlook

Herbicide application is an inefficient industrial product as large proportions of it ends up in the environment. One problematic issue is herbicide-resistant weeds, which occurs in part due to its prolonged use. It is envisaged that herbicide resistance problems will persist due to farmers’ reluctance in taking measures to prevent it. For example, farmers still use high herbicide dosages, multiple types of herbicides, and fail to execute environmentally friendly herbicide management programs. So, the status-quo persists as herbicide application remains a central component of modern arable farming because it continues to increase crop productivity and yield despite its flaws. The ongoing challenge is to design an effective herbicidal agent that on the one hand adheres to environmental regulations, while on the other hand reduces public health concerns about adverse environmental and human health effects. The problems arising from herbicide-resistance have been at the forefront of research in many countries worldwide, as it is a formidable issue. As herbicide application is envisaged to remain a standard part of agricultural practice, the research will continue to focus on objectives such as synthesizing herbicides with improved eco-friendly ratings. Additionally, more field evaluations need to be carried out to determine bioherbicide efficiency (as a more direct technique to complement chemical-based herbicide application) taking into consideration active components, crop selectivity, and other related parameters. Herbicide manufacturers and researchers would do well to heed farmer input, as they are the direct beneficiaries of herbicide development. Farmers have been calling for a herbicide formulation that deviates from the common ready-to-use formulation. This would ensure versatility and wider herbicide applications in agricultural settings (from economical perspective) as it allows for custom formulations. All of these efforts aim to improve crop yield, eliminating invasive vegetation threats and ensuring optimum plant growth while ultimately reducing the adverse effects of herbicides on human health and ecosystems.

## Data Availability

Data sharing is not applicable to this article as no new data were created or analyzed in this study. All images were obtained from free-access images on freepik.com and Wikimedia Commons.

## Highlights

- Understanding the source and classification of herbicides is important for practitioners involved in weed management programs.
- The adverse effects of herbicides on human public health are discussed.
- The physico-chemical, biological, and chemical methods, including advanced oxidation processes (AOPs) for herbicide removal are reviewed.
- The advantages and limitations of alternative, green, and sustainable treatment methods are summarized.
